# Supporting the Mind in Space: Psychological Tools for Long-Duration Missions

**DOI:** 10.2196/66626

**Published:** 2024-10-09

**Authors:** Francesco Pagnini

**Affiliations:** 1 Department of Psychology Università Cattolica del Sacro Cuore Milan Italy

**Keywords:** space psychology, astronauts, psychotherapy, isolated and confined environment, mindfulness, relaxation, mind-body

## Abstract

The psychological well-being of astronauts is becoming just as vital as their physical and technical readiness as space missions extend into deep space. Long-duration missions pose unique challenges, such as isolation, confinement, communication delays, and microgravity, which can significantly affect mental health and cognitive performance. This commentary discusses the need for innovative mental health support systems, including automated psychotherapy, as well as Earth-based training methods like mindfulness and relaxation techniques, to address the psychological demands of space travel. By integrating these approaches into pre-mission preparation and in-flight routines, astronauts can develop self-regulation strategies to manage stress, improve focus, and enhance emotional resilience. Automated psychotherapy available 24-7 provides real-time confidential support when communication with Earth is delayed. As space exploration moves forward, the success of missions will depend not only on technological advancements but also on the development of psychological countermeasures that prioritize mental health alongside physical well-being. This paper emphasizes the importance of continued research and collaboration to refine and test these tools in analog environments, ensuring astronauts are mentally and emotionally prepared for the challenges of space.

## Introduction

The manuscript “Automated Psychotherapy in a Spaceflight Environment: Advantages, Drawbacks, and Unknowns,” written by Smith [1], explores the potential of automated psychotherapy to address the mental health challenges faced by astronauts during space missions. This technique could be highly relevant for human space exploration, particularly in overcoming delayed communication issues in long-distance missions. However, further research is needed in this field, which represents a new frontier in both space exploration and mental health care.

## Psychological Aspects of Space Exploration

As humanity pushes further into space, the psychological well-being of astronauts becomes as critical as the technical aspects of a mission [1]. Long-duration missions, including those on future Mars and lunar habitats, present unique challenges beyond just the physical demands [2]. Astronauts face months, even years, of isolation, confinement, communication delays, and microgravity in an extreme environment that is, from multiple perspectives, “hostile” to human life. These factors can impact mental health; cognitive performance; and, ultimately, mission success [3].

Astronauts are highly trained individuals, but they are still human. Prolonged isolation and distance from Earth, coupled with the stress of living and working in space, can lead to undesirable psychological reactions and reduced cognitive function [4,5]. As missions become longer and more distant, space agencies need to consider not only the physical but also the psychological needs of astronauts to ensure that they can function at their best.

## Psychological Support in Space

Providing psychological support during space missions is not a new concept [6], but as missions extend into deep space, the challenges become more complex. Traditionally, astronauts have had access to Earth-based psychologists via private communications. Private consultations and ongoing support can help manage interpersonal tensions, alleviate feelings of isolation, and address stress in real time. While this has worked well for missions in low earth orbit, such as those aboard the International Space Station, long communication delays in deep space are expected to make this system less effective [2]. For example, a Mars mission could experience a communication delay of up to 30 minutes each way. In such cases, astronauts need tools that allow for real-time mental health support, including the possibility of an automated psychotherapy system. This is also where pre-mission psychological training and on-demand support systems come into play. These include resilience training, team dynamics workshops, and tailored psychological tools that astronauts can access during the mission.

## Mindfulness and Relaxation: Key Tools for Managing Stress

One promising approach to managing stress during long missions is through mindfulness and relaxation techniques [7]. These practices have been shown to substantially reduce stress, enhance focus, and improve emotional regulation, making them valuable tools for astronauts facing long-term isolation and confinement. Mindfulness encourages individuals to stay present and manage stress without judgment, which can be particularly helpful in an environment where stressors are constant and inevitable. Astronauts trained in mindfulness techniques are expected, according to subject matter experts, to handle stress more effectively, maintaining mental clarity and decision-making abilities even in high-pressure situations [8]. Recently, mindfulness disposition has been demonstrated as a protective factor against stress in an analog environment, the Concordia base in Antarctica [9].

Relaxation training, such as diaphragmatic breathing and progressive muscle relaxation, can also play an important role. These techniques can reduce physiological stress, promote better sleep, and help astronauts manage their emotional responses to challenging situations. Given the stressful and sometimes monotonous environment of space, these practices offer simple yet powerful ways to maintain both mental and physical well-being [7].

By integrating mindfulness and relaxation into astronauts’ training, space agencies can equip them with effective self-regulation tools to help manage the ongoing psychological demands of space missions. These methods are not only noninvasive but also easy to practice, making them ideal for space, where time and resources are limited.

## The Role of Automated Psychotherapy in Space

With communication delays limiting real-time support from Earth, automated psychotherapy offers a crucial solution for mental health care during space missions. These systems can provide astronauts with immediate access to cognitive behavioral therapy and other interventions tailored to their specific needs and the unique stresses of space travel. One of the most compelling aspects of Smith’s [1] manuscript is the focus on how automated psychotherapy could overcome the challenges of delayed communication in deep space missions. This is particularly relevant as human space exploration moves toward Mars and beyond, where real-time communication with Earth will no longer be possible. Smith’s [1] work provides a strong foundation for understanding the psychological complexities of long-duration missions, particularly the potential for automated systems to fill gaps in mental health support when traditional Earth-based therapy is not feasible.

Automated psychotherapy tools also offer additional advantages. They are available 24-7, offering astronauts confidential on-demand support. This is particularly useful for addressing sensitive issues that astronauts might not feel comfortable discussing with crewmates or mission control. These systems can guide astronauts through therapeutic exercises, helping them manage anxiety, stress, or feelings of isolation as they arise. Furthermore, automated systems can be integrated with other mental health tools, such as mindfulness and relaxation practices, creating a comprehensive mental health support network. This combination of tools ensures that astronauts can address both immediate psychological needs and long-term mental health maintenance without relying on Earth-based professionals. As these systems are refined, they have the potential to become a critical component of mental health care for long-duration missions.

## Conclusion

As space exploration advances into longer, more isolated missions, the psychological and human aspects of space travel become increasingly critical. The challenges of isolation, stress, and confinement in space demand innovative solutions that go beyond traditional mental health support [2].

The integration of automated psychotherapy, as well as newly developed training techniques (eg, mindfulness and relaxation training), offer a proactive and effective approach to safeguarding astronaut mental health. By providing astronauts with these essential tools, space agencies can ensure that crews are not only physically prepared but also mentally resilient, capable of adapting to the extreme demands of long-duration missions. The success of future space exploration, however, will depend on our collective commitment to supporting the psychological well-being of astronauts. The development, testing, and refinement of these psychological tools in analog environments are imperative. As we advance into the next phase of space exploration, space agencies, researchers, and innovators need to work together to ensure that mental health receives the same attention as physical and technical preparation, helping astronauts thrive on their journeys into the unknown.
